# The English national cohort study of flooding and health: cross-sectional analysis of mental health outcomes at year one

**DOI:** 10.1186/s12889-016-4000-2

**Published:** 2017-01-28

**Authors:** Thomas David Waite, Katerina Chaintarli, Charles R. Beck, Angie Bone, Richard Amlôt, Sari Kovats, Mark Reacher, Ben Armstrong, Giovanni Leonardi, G. James Rubin, Isabel Oliver

**Affiliations:** 1 Field Epidemiology Training Programme, Public Health England, Bristol, UK; 2 European Programme for Interventional Epidemiology Training, Stockholm, Sweden; 3 Field Epidemiology Service, Public Health England, 2 Rivergate, BS1 6EH Bristol, UK; 4 School of Social and Community Medicine, University of Bristol, Bristol, UK; 5 Centre for Radiation, Chemicals and Environmental Hazards, Public Health England, 133-155 Waterloo Road, Chilton, SE1 8 UG London UK; 6 Emergency Response Department, Public Health England, Porton Down, Wilts, SP4 0JG Salisbury UK; 7 NIHR Health Protection Research Unit in Environmental Change and Health at the London School of Hygiene and Tropical Medicine, 15-17 Tavistock Place, London, WC1H 9SH UK; 8 Department of Psychological Medicine, Weston Education Centre, King’s College London NIHR Health Protection Research Unit in Emergency Preparedness and Response, Cutcombe Road, SE5 9RJ London, UK; 9 NIHR Health Protection Research Unit in Evaluation of Interventions at the University of Bristol, Bristol, UK; 10 Field Epidemiology Service, Public Health England, Cambridge, UK; 11 Public Health England, Chilton, Didcot, OX11 ORQ Oxon UK

## Abstract

**Background:**

In winter 2013/14 there was widespread flooding in England. Previous studies have described an increased prevalence of psychological morbidity six months after flooding. Disruption to essential services may increase morbidity however there have been no studies examining whether those experiencing disruption but not directly flooded are affected.

The National Study of Flooding and Health was established in order to investigate the longer-term impact of flooding and related disruptions on mental health and wellbeing.

**Methods:**

In year one we conducted a cross sectional analysis of people living in neighbourhoods affected by flooding between 1 December 2013 and 31 March 2014. 8761 households were invited to participate. Participants were categorised according to exposure as flooded, disrupted by flooding or unaffected.

We used validated instruments to screen for probable psychological morbidity, the Patient Health Questionnaire (PHQ 2), Generalised Anxiety Disorder scale (GAD-2) and Post Traumatic Stress Disorder (PTSD) checklist (PCL-6).

We calculated prevalence and odds ratios for each outcome by exposure group relative to unaffected participants, adjusting for confounders.

**Results:**

2126 people (23%) responded. The prevalence of psychological morbidity was elevated amongst flooded participants ([*n* = 622] depression 20.1%, anxiety 28.3%, PTSD 36.2%) and disrupted participants ([*n* = 1099] depression 9.6%, anxiety 10.7% PTSD 15.2%).

Flooding was associated with higher odds of all outcomes (adjusted odds ratios (aORs), 95% CIs for depression 5.91 (3.91–10.99), anxiety 6.50 (3.77–11.24), PTSD 7.19 (4.33–11.93)).

Flooded participants who reported domestic utilities disruption had higher odds of all outcomes than other flooded participants, (aORs, depression 6.19 (3.30–11.59), anxiety 6.64 (3.84–11.48), PTSD 7.27 (4.39–12.03) aORs without such disruption, depression, 3.14 (1.17–8.39), anxiety 3.45 (1.45–8.22), PTSD 2.90 (1.25–6.73)). Increased floodwater depth was significantly associated with higher odds of each outcome.

Disruption without flooding was associated with borderline higher odds of anxiety (aOR 1.61 (0.94–2.77)) and higher odds of PTSD 2.06 (1.27–3.35)) compared with unaffected participants. Disruption to health/social care and work/education was also associated with higher odds of psychological morbidity.

**Conclusions:**

This study provides an insight into the impact of flooding on mental health, suggesting that the impacts of flooding are large, prolonged and extend beyond just those whose homes are flooded.

## Background

Floods have significant health impacts. In the last decade [1] over 1000 people were killed by floods and 5 · 6 million more were affected in the World Health Organization (WHO) European region.

Immediate impacts from flooding are usually due to injuries, infections, chemical hazards, and disruption to health services; the longer term effects are less well understood and may arise from displacement, damage to homes, and loss of domestic utilities [2].

Research suggests that climate and population change will result in more frequent flooding in the United Kingdom (UK) [3]. River flooding may affect 250,000–400,000 additional people across Europe by the 2080s with the UK and Central Europe among the most severely affected [2].

The impact of flooding on physical health includes injury and infectious disease, particularly gastrointestinal illness [4]. In high income countries such as the UK floods cause few immediate deaths; it is estimated that mental health problems are responsible for 80% of all Disability Adjusted Life Years (DALYs) attributable to floods in the UK [5]. A recent systematic review concluded that there is a shortage of research into the mental health effects of fluvial flooding, as opposed to coastal, tsunami or hurricane related [6]. Previous studies have described an increased prevalence of psychological morbidity in the short term [7, 8]; individuals affected by floodwater in the home were found to be two to five times more likely to suffer psychological morbidity three to six months after flooding compared to unaffected individuals. Disruption to essential services increased such morbidity [7].

The time course of the mental health impact of flooding is not well understood. Previous epidemiological studies undertook surveys up to six months after flooding. Qualitative evidence suggests that the impact on mental health may last for years after flooding [8], but this has not been quantitatively described using longitudinal data.

In the winter of 2013/14 there was widespread river, coastal and surface water flooding in England. Approximately 6500 addresses were flooded; flood warnings were issued to 2.5 million properties [9]. Many people whose homes were not flooded suffered significant disruption including interruption to infrastructure and domestic utilities. Many different flooding events occurred over at least four months, including river, coastal and surface water flooding.

The National Study of Flooding and Health was established in order to investigate the longer term impact of flooding and related disruption on mental health and wellbeing, to help direct preventive and follow up actions, and to reduce harm from future flooding. Uniquely, we will follow up participants who suffered disruption in the absence of flooding as well as flooded individuals, beginning 12 months after flooding with annual follow up thereafter in order to address two research gaps; providing descriptions of both the persistence of symptoms of psychological morbidity beyond six months, and the impact on those whose lives were disrupted by the flooding but who did not personally have floodwater in their homes. Our objectives in the first year of the study were to:

1. Describe the prevalence of probable depression, anxiety, and post-traumatic stress disorder (PTSD) at one year among individuals exposed to flooding or disruption from flooding compared to those unexposed
2. Identify personal and sociodemographic characteristics associated with psychological morbidity.

## Methods

### Study design

A cross sectional analysis one year after flooding was undertaken, nested within a planned cohort study. All participants lived in neighbourhoods affected by flooding in the south of England between 1 December 2013 and 31 March 2014.

We obtained lists of those postcode areas affected by flooding held by local authorities (Gloucestershire, Wiltshire, Surrey, Sedgemoor, South Somerset, and Tonbridge and Malling). These were gathered for emergency response purposes and varied in content and format. We used the Royal Mail Postcode Address File (PAF), which is a complete listing of properties in the United Kingdom, to generate a list of the full addresses of all residential properties in each postcode area. [10] The PAF was accessed using Addressbase, an Ordnance Survey product which allows each unique address to be classified as residential or commercial.

### Study population

A recruitment pack including a questionnaire was sent to each household address in the postcode areas identified. In Surrey, due to larger numbers, recruitment packs were sent to all addresses supplied by the local authority as well as 4110 addresses chosen at random from the list of 7205 identified by Addressbase. Recruitment packs were distributed by post in January 2015. All adults aged over 18 residing at addresses to which recruitment packs were sent were invited to participate; a cover letter advised households requiring more than one questionnaire to either request additional questionnaires by telephone or to complete the survey online.

### Data collection

We invited participants to return the questionnaire by post or online. The 36-item questionnaire included a bespoke 19 item exposure assessment. All participants were asked if their home had been flooded as well as questions to ascertain if they had experienced a range of disruptions and potential secondary stressors (such as aspects of insurance).

We followed the definition of flooding used in previous studies for comparability [7, 11]. Participants were allocated to one of three categories:

- Unaffected participants, who reported no flooding or disruption to their lives from flooding in their area
- Disrupted participants, defined as those who reported no floodwater in the liveable rooms of their home and at least one of the following disruptions:
  - Evacuation
  - Flooding of non-liveable areas, garages, gardens or the street
  - Interruption to household utilities (electricity, gas, oil, water, drainage, septic tank)
  - Loss of communications (postal or telecommunications)
  - Interruption to health or social care access, in or away from the home
  - Difficulty accessing work, own or children’s education
  - Interruption to other amenity e.g. getting to shops or social activities
- Flooded participants, who reported floodwater in at least one liveable room of their home

Participants who reported flooding were asked to answer questions about duration, depth and consequences including damage to the home and displacement. We collected demographic data including sex, date of birth, ethnicity, marital status, household composition and tenure, education, employment, and the presence of any limiting long term illness.

We used validated instruments and cut-off scores employed in clinical practice to screen for symptoms suggestive of probable mental health outcomes; these included the Patient Health Questionnaire (PHQ-2) for depression, Generalised Anxiety Disorder scale (GAD-2) for anxiety and Post Traumatic Stress Disorder (PTSD) checklist (PCL-6) for PTSD. Cut-off scores were ≥3 for PHQ-2/GAD-2 and ≥14 for PCL-6 [12–14]. The questionnaire is attached as an appendix/online resource.

Lower Super Output Area (LSOA) deprivation score using the Index of Multiple Deprivation (IMD) was assigned to each participant using postcode of residence. Participants were then divided into five groups, based on the all-England LSOA IMD rank [15].

### Statistical methods

Prevalence of each mental health outcome was calculated as a percentage of the total for each exposure category. We calculated odds ratios for each mental health outcome in each exposed group relative to the unaffected group, adjusting by logistic regression for those variables considered *a priori* as potential confounders. These were age group, sex, local authority (sampling design strata), ethnicity, marital status, education level, employment, and deprivation score. All these a priori confounders were included in the model as categorical variables, regardless of significance. Odds ratios were considered to show a significant (p < 0.05) difference from the unaffected group if the 95% confidence interval did not include 1. In analyses yielding odds ratios for ordered sub-groups of exposure (e.g. by depth of flooding), we carried out a Wald test for the trend over those odds ratios, ignoring the reference (unaffected) group.

Participants who provided inadequate data to allow exposure categorisation were excluded from further analysis. Participants who did not complete an outcome instrument were excluded from analysis of that measure only. Data were entered using Epidata (Epidata Association, Denmark). The online questionnaire was designed using SelectSurvey (ClassApps, USA). Analyses were performed using Stata 12 (Statacorp, USA).

## Results

We sent recruitment packs to 8761 households. Responses were received from 2126 participants from 2014 households (household response rate 23%). 251 participants (12.5%) responded online. Participant characteristics by exposure group are shown in supplementary information.

Exposure classification could be assigned for 2006 participants. In total, 285 (14.2%) were classified as unaffected, 622 (31.0%) as flooded and 1099 (54.8%) as disrupted.

The prevalence of probable depression, anxiety and PTSD was elevated amongst both flooded and disrupted participants, compared with the unaffected group (Table 1). Adjusted odds of probable psychological morbidity were six to seven times higher for flooded than unaffected participants and 1.5–2.0 times higher for disrupted participants (Table 2).

**Table 1:** Prevalence of mental health outcomes by exposure group

Outcome	Overall cohort	Unaffected	Exposure group	
			Disrupted	Flooded
Probable depression	250/1929 (12.6%)	16/278 (5.8%)	102/1058 (9.6%)	125/593 (20.1%)
Probable anxiety	300/1927 (15.6%)	18/278 (6.5%)	113/1052 (10.7%)	169/597 (28.3%)
Probable PTSD	396/1925 (20.6%)	22/278 (7.9%)	160/1056 (15.2%)	214/591 (36.2%)

**Table 2:** Crude and adjusted odds ratios (OR) of mental health outcome amongst disrupted and flooded participants compared with unaffected participants

Outcome	Crude OR (95% CI)	aOR* (95% CI)
Probable depression		
Disrupted	1.75 (1.01–3.01)	1.56 (0.88–2.76)
Flooded	4.37 (2.54–7.52)	5.91 (3.17–10.99)
Probable Anxiety		
Disrupted	1.74 (1.04–2.91)	1.61 (0.94–2.77)
Flooded	5.70 (3.43–9.49)	6.50 (3.77–11.24)
Probable PTSD		
Disrupted	2.08 (1.30–3.31)	2.06 (1.27–3.35)
Flooded	6.61 (4.14–10.53)	7.19 (4.33–11.93)

*Adjusted odds ratios are adjusted for age, sex, pre-existing illness, deprivation, local authority, ethnicity, marital, education and employment statuses

### Association between psychological morbidity and floodwater (Table 3)

**Table 3:** Association between mental health outcomes and nature of flooding in liveable rooms.

Explanatory variable		Outcome								
	Total	Depression			Anxiety			PTSD		
		n	aOR	(95% CI)	n	aOR	(95% CI)	n	aOR	(95% CI)
Unaffected	285	16	1.00		18	1.00		22	1.00	
Flooded	622	125	5.91	(3.17–10.99)	169	6.50	(3.77–11.24)	214	7.19	(4.33–11.93)
Depth										
Depth <30 cm	376	59	*4.58	(2.38–8.80)	86	*5.28	(3.00–9.32)	110	*5.72	(3.39–9.63)
30–100 cm	191	51	*8.48	(4.21–17.10)	66	*8.97	(4.86–16.57)	85	*10.12	(5.74–17.87)
>100 cm	27	10	*14.71	(4.45–48.62)	11	*11.40	(3.93–33.08)	16	*17.79	(6.33–50.01)
Flood duration										
<24 h	108	10	^+^2.60	(1.06–6.39)	24	5.53	(2.73–11.18)	30	5.39	(2.81–10.35)
24 h–7 days	199	43	^+^5.82	(2.92–11.58)	59	7.12	(3.87–13.04)	71	7.04	(4.01–12.36)
8 days–2weeks	72	16	^+^6.50	(2.82–14.97)	16	4.28	(1.97–9.29)	23	6.11	(3.01–12.39)
>2weeks	156	36	^+^5.99	(2.87–12.47)	45	5.62	(2.94–10.73)	63	7.72	(4.26–14.03)
Now able to use liveable rooms										
Able	351	74	^+^4.65	(2.42–8.93)	109	*5.38	(3.14–9.77)	139	**7.08	(4.18–11.99)
Not yet able	27	10	^+^11.43	(3.86–33.85)	13	*15.47	(5.82–41.10)	19	**27.12	(9.60–76.67)
Evacuation										
Flooded, not evacuated	225	132	4.14	(2.16–7.93)	153	^+^4.20	(2.38–7.42)	196	**4.05	(2.38–6.88)
Flooded and evacuated	397	102	5.81	(3.03–11.11)	136	^+^6.16	(3.51–10.82)	184	**7.36	(4.37–12.39)
Displacement										
≤14 days	28	13	10.73	(3.80–30.34)	22	10.93	(4.31–27.73)	22	12.40	(4.92–31.22)
14–180 days	100	18	3.90	(1.70–8.95)	32	6.51	(3.28–12.96)	37	7.22	(3.78–13.77)
>180 days	189	39	5.18	(2.56–10.51)	56	6.31	(3.41–11.69)	80	8.60	(4.87–15.19)
Moving out due to flooding or damage										
Flooded but did not move out	189	88	4.90	(2.44–9.85)	104	4.77	(2.58–8.82)	136	**4.27	(2.41–7.58)
Flooded and moved out	424	102	6.19	(3.25–11.79)	145	6.94	(3.96–12.16)	185	**8.32	(4.96–13.98)
Moved back in										
Has moved back in	380	86	5.61	(2.92–10.78)	125	6.49	(3.68–11.43)	161	8.13	(4.81–13.73)
Planning to move back in	39	13	9.04	(3.34–24.49)	18	9.83	(4.07–23.74)	20	8.66	(3.69–20.32)
Not planning to move back in	9	5	34.24	(7.65–153.27)	4	13.52	(3.18–57.45)	5	14.51	(3.47–60.66)

All comparisons made with unaffected respondents Adjusted odds ratios are adjusted for age, sex, pre-existing illness, deprivation, local authority, ethnicity, marital, education and employment statuses; Odds ratios with a 95% CI that excludes 1.0 imply significant elevation at *p* < 0.05 Significance of trend across flooded subgroups is indicated by + at *p* < 0.1, *at *p* < 0.05 and **at *p* < 0.01

An increased effect for increased depth of flooding was observed for each outcome. The adjusted odds of probable PTSD amongst flooded participants increased from approximately 4.5 times higher for floodwater depth <30 cm to nearly 15 times higher for depth >100 cm (*p* < 0.01). A similar increase was seen for probable depression and anxiety. Participants reporting floodwater in the home for more than 24 h had slightly higher odds of adverse outcomes, in particular depression, than those flooded for a shorter period. Beyond 24 h there was no evidence for increasing odds of probable psychological morbidity with increased duration of flooding.

The small number (27) of participants who had not regained use of all liveable rooms of their home had markedly higher odds of all outcomes when compared with either unaffected participants or those who had been flooded and had regained the use of all rooms at the time of answering the questionnaire.

Those flooded and also evacuated had six to seven times higher odds of probable psychological morbidity than unaffected participants and somewhat higher odds than those flooded but not evacuated, significantly so for PTSD (*p* < 0.01). Overall, those who were flooded and who moved out for any duration had over eight times higher odds of probable PTSD than unaffected participants and significantly higher odds than those who were flooded but did not leave their home (*p* < 0.01). The nine participants who had been displaced and did not plan to move back had particularly high odds of all outcomes, though given the small numbers, estimates were imprecise and differences with other flooded persons not significant.

### Association between probable psychological morbidity and disruptions (Tables 4 and 5)

**Table 4:** Association between mental health outcomes and disruptions amongst those who experienced flooding in a liveable room

		Outcome								
	N	Depression			Anxiety			PTSD		
Explanatory variable		n	aOR	(95% CI)	n	aOR	(95% CI)	n	aOR	(95% CI)
Unaffected	285	16	1.00		18	1.00		22	1.00	
Flooded	622	125	5.91	(3.17–10.99)	169	6.50	(3.77–11.24)	214	7.19	(4.33–11.93)
Disruption to utilities										
Flooded but did not lose any utilities^a^	32	8	3.14	(1.17–8.39)	10	^+^ 3.45	(1.45–8.22)	10	* 2.90	(1.25–6.73)
Flooded and did lose a utility	510	110	6.19	(3.30–11.59)	148	^+^ 6.64	(3.84–11.48)	188	* 7.27	(4.39–12.03)
Disruption to health and social care										
Flooded but did not lose access^a^	45	13	5.55	(2.31–13.35)	17	6.56	(3.04–14.15)	17	^+^ 5.22	(2.46–11.06)
Flooded and lost	139	30	5.06	(2.43–10.54)	46	6.81	(3.63–12.78)	60	^+^ 9.62	(5.31–17.43)
Disruption to communications										
Flooded but did not lose access^a^	324	61	4.24	(2.25–7.99)	88	4.79	(2.76–8.32)	105	5.09	(3.04–8.50)
Flooded and lost	260	59	5.73	(2.95–11.15)	76	5.95	(3.32–10.66)	99	6.89	(4.00–11.84)
Loss of access to work or education										
Flooded, did not lose access^a^	87	15	4.05	(1.80–9.18)	25	6.06	(3.01–12.18)	30	5.33	(2.79–10.19)
Flooded and did lose access	228	52	6.34	(3.19–12.60)	70	7.43	(4.06–13.59)	91	7.74	(4.43–13.51)
Loss of other access (e.g. social activities)										
Flooded, did not lose access^a^	281	56	4.45	(2.33–8.48)	78	4.98	(2.83–8.74)	94	* 4.83	(2.87–8.14)
Flooded and did lose access	231	50	5.61	(2.85–11.02)	72	6.85	(3.80–12.30)	90	* 8.00	(4.62–13.83)

^a^excludes those who responded not applicable/do not routinely access this service/utility or did not need to during the study period

Significance of inter-group heterogeneity across flooded subgroups is indicated by + at *p* < 0.1, * at *p* < 0.05 and ** at *p* < 0.01

All comparisons made between flooded and unaffected respondents. Adjusted odds ratios are adjusted for age, sex, pre-existing illness, deprivation, local authority, ethnicity, marital, education and employment statuses

**Table 5:** Association between mental health outcomes and disruptions in those who did not experience flooding in a liveable room

Outcome										
	N	Depression			Anxiety			PTSD		
Explanatory variable		n	aOR	(95% CI)	n	aOR	(95% CI)	n	aOR	(95% CI)
Unaffected	285	16	1.00		18	1.00		22	1.00	
All disrupted	1099	102	1.56	(0.88–2.76)	113	1.61	(0.94–2.77)	160	2.06	(1.27–3.35)
Disruption to utilities										
Disrupted but did not lose any utilities^a^	209	24	1.62	(0.81–3.24)	22	1.40	(0.71–2.75)	34	1.96	(1.08–3.54)
Disrupted and did lose a utility	623	59	1.58	(0.86–2.88)	65	1.59	(0.90–2.80)	99	2.30	(1.37–3.83)
Disruption to health and social care										
Disrupted but did not lose access^a^	131	16	^+^ 1.70	(0.79–3.66)	17	^+^ 1.56	(0.75–3.24)	32	3.05	(1.65–5.65)
Disrupted and lost	271	46	^+^ 3.28	(1.73–6.19)	44	^+^ 2.86	(1.55–5.27)	57	3.50	(2.01–6.10)
Disruption to communications										
Disrupted but did not lose access^a^	864	80	1.41	(0.79–2.52)	96	1.58	(0.92–2.72)	125	^+^ 1.86	(1.14–3.04)
Disrupted and lost	154	19	2.21	(1.05–4.64)	19	2.06	(1.00–4.21)	30	^+^ 2.95	(1.57–5.51)
Loss of access to work or education										
Disrupted, did not lose access^a^	160	8	** 0.72	(0.28–1.83)	13	* 1.02	(0.47–2.24)	24	1.83	(0.96–3.50)
Disrupted and did lose access	392	44	** 2.20	(1.15–4.23)	56	* 2.30	(1.27–4.17)	70	2.70	(1.56–4.67)
Loss of other access (e.g. social activities)										
Disrupted, did not lose access^a^	466	33	** 1.00	(0.52–1.91)	42	** 1.11	(0.61–2.02)	61	** 1.51	(0.89–2.57)
Disrupted and did lose access	443	57	** 2.53	(1.37–4.68)	57	** 2.29	(1.28–4.10)	81	** 2.98	(1.76–5.06)
Evacuation										
Disrupted, not evacuated	923	90	1.49	(0.84–2.65)	97	1.49	(0.87–2.56)	131	1.80	(1.10–2.93)
Disrupted, did evacuate	176	19	1.76	(0.83–3.70)	23	1.95	(0.98–3.90)	35	3.08	(1.67–5.66)

^a^excludes those who responded not applicable/do not routinely access this service/utility or did not need to during the study period

Significance of inter-group heterogeneity across disrupted subgroups is indicated by + at p < 0.1, * at *p* < 0.05 and ** at *p* < 0.01

All comparisons made between disrupted and unaffected participants, excluding flooded participants. Adjusted odds ratios are adjusted for age, sex, pre-existing illness, deprivation, local authority, ethnicity, marital, education and employment statuses

Flooded participants who reported disruption to domestic utilities had six to seven times higher odds of probable psychological morbidity than unaffected participants, higher than flooded participants who did not report such disruptions, though only significantly so (*p* < 0.05) for probable PTSD. Disruption or loss of access to health or social care was associated with over nine times higher odds of probable PTSD than unaffected participants and borderline raised odds compared to flooded participants who did not report such disruptions (Table 4).

There were only modestly increased odds of psychological morbidity in flooded participants who reported disruption to work, education or communications compared with those who were flooded and did not report such disruptions, and none different significantly (*P* < 0.05). Amongst disrupted participants who did not report flooding, disruption to health and social care was associated with significantly increased odds of psychological morbidity compared with unaffected participants and borderline significantly increased odds of depression and anxiety compared with those who reported other forms of disruption (Table 5). Disruption of access to work and education was also associated with increased odds of psychological morbidity compared with both unaffected participants and those who reported other forms of disruption. Significantly increased odds of all outcomes were also observed for loss of access to shops or social activities.

## Discussion

Our study has shown that both flooding and certain forms of disruption from flooding are associated with elevated high risk of psychological morbidity. This is the first study to have examined the impact on people living in areas which experienced flooding but who did not have floodwater in their own homes and the first to examine associations between psychological morbidity and particular disruptions.

Even amongst those who did not have floodwater in their homes, loss of access to health or social care, work, education or social activities were associated with elevated odds of psychological morbidity a year after flooding when compared with either unaffected participants or those who experienced other forms of disruption.

For disrupted participants, we found that loss of access to health and social care was associated with the greatest increase in odds of psychological morbidity; loss of access to employment, education and other activities were also significant.

For this cross sectional analysis, data was collected via self-completed questionnaires and was subject to several potential biases. There are no national or local registers of flooded homes; we thus needed to make extensive efforts to identify a suitable population. We wrote to individual postcode areas rather than entire administrative districts (such as local authorities). This means that we cannot compare the characteristics of our respondents with the population data from, for example, the census in order to check the representativeness of our respondents. We could not know the number of unaffected, disrupted and flooded households in each area, hence we sought a wide range of self-reported exposure information. We examined the impact of flooding on mental health in several areas of the south of England. On this occasion, affected areas included some of the most affluent parts of the country. We anticipated this scenario and designed our analysis to allow for this; in adjusting for age, sex, pre-existing illness, deprivation, local authority, ethnicity, marital, education, and employment statuses we have taken into account confounding from these factors. The very low numbers of participants from the most deprived areas and who were from ethnic minority groups affects generalisability of our findings to these groups.

We aimed to identify sufficient numbers of participants who had experienced disruption and flooding and in doing so wrote to households, inviting all members to participate. A number of limitations are inherent in this approach; the responders were self selecting and, because we only sent one questionnaire to each household it would have been more difficult for additional household members to participate.

It was possible that places which experienced only limited flooding or minor disruption were not known to local authorities. Similarly, those who experience flooding regularly may not have sought help from the local authority.

It was notable that smaller numbers of unaffected respondents participated than disrupted or flooded respondents, potentially because they were less interested in the topic of the study. Previous research suggests topic interest may have resulted in people who were particularly adversely affected by the flooding being more likely to participate [16–18], potentially inflating our estimates of the association between flooding and psychological morbidity. It is also possible that unaffected people with symptoms of potential psychological morbidity were less likely to respond than people with symptoms who had experienced flooding or disruption, which would also have exaggerated the effect size.

An alternative interpretation is that, since avoidance is a symptom of many forms of psychological morbidity, sufferers may have been more likely to avoid completing the questionnaire which constituted an unpleasant reminder of the floods, leading to an underestimate of prevalence in all groups. The prevalence of psychological morbidity amongst unaffected respondents was in keeping with previous studies [7]. By way of context, it has been estimated that the prevalence of mixed anxiety and depressive disorder in England is approximately 8.8% with prevalence of generalised anxiety disorder of approximately 4.4% [19]. Those people who were worst affected by flooding may have been missing from our sample, if they were still displaced from their homes at the time our questionnaires were sent out. As this group might be expected to have greater risk of psychological morbidity, this may also have led to underestimations in the effect size.

Interpretation of specific comparisons must be cautious. Although the number and strength of the associations are such that most are very unlikely to be due to chance, there were many statistical tests of association undertaken so that that some “significant” ones might be chance findings. Further work is required to understand and validate these findings which have been quantified here for the first time. It is logical that amongst flooded participants, increased depth of flooding would be associated with increased psychological morbidity. The observed effect for this variable was plausible and gave us confidence that our study provided a true representation of the impact of flooding on mental health. We found a plateau effect in the odds of psychological morbidity with increasing flood duration which requires further exploration. Potential explanatory factors include community support, the actions of emergency services or other mitigating factors after the initial flood event. Odds ratios for disruption type are not mutually adjusted (i.e. for types of disruption) and thus we cannot be sure whether observed associations with specific disruptions reflect individuals also experiencing another form of disruption. This is particularly important when interpreting more marginally elevated odds of morbidity.

Finally, our outcome measurement tools are used as clinical screening tools; whilst they indicate probable presence of disease, they are not clinically diagnostic.

## Conclusions

Rates of psychological morbidity among flooded participants one year after flooding were between 20.1% and 36.2%. These rates are comparable to a previous UK study [7] at three to six months post flooding. An elevated risk was also observed amongst those whose homes were not flooded but their lives were nevertheless affected. Flooding is likely to exacerbate the challenge of poor mental health in many communities. Commissioners and providers of primary, community and mental health services as well as emergency planners should prepare for an increased need for services in areas affected, or likely to be affected, by flooding.

Levels of depression and PTSD amongst people whose homes were flooded were comparable to the rates of clinically diagnosable disorder among the members of the public involved in major incidents. For example, 30–40% of those closest to the site of a terrorist attack have been found to have clinically diagnosable mental health disorder [20]. National Institute of Health and Care Excellence (NICE) guidelines, currently under revision, for the management of PTSD following major disasters cautiously recommend that consideration be given to proactive screening of victims for mental health disorder, followed by referral for appropriate evidence-based treatment where required [21]. While flooding is not explicitly referred to in this guidance, the high rates of disorder identified in this study suggest that proactive approaches to identify and support people affected by flooding could be valuable.

Amongst those whose homes were flooded, factors including increased depth of floodwater, loss of a utility or more than 24 h of flooding of the home were associated with increased odds of psychological morbidity. This may reflect severity of flooding and serve as useful markers to help identify and support people at risk of psychological morbidity and suggests that limiting ingress of water to flooded homes may prevent greater mental health impacts on occupants in addition to the obvious limitation of damage to the home.

Disruption to health and social care access increased the risk of psychological morbidity for those who usually use these services and either had floodwater in their homes or were disrupted in other ways. This suggests that those involved in response and recovery should identify and support these individuals and prioritise reinstatement of services.

Amongst those disrupted by flooding but without floodwater in the home, disruption to work and education was a more important predictor of psychological morbidity than in those who were flooded, possibly because they were not already dealing with the stress of damage to the home. Reinstating access to these activities may thus reduce mental health impacts as well as supporting community recovery more generally. This study provides an insight into the impact of flooding on probable depression, anxiety and PTSD. These associations suggest that the mental health impacts of flooding are prolonged and extend beyond just those who are flooded. This represents a key finding for providers of these services as well as agencies who respond to emergencies.

## Abbreviations

aOR: Adjusted Odds Ratio
IMD: Index of Multiple Deprivation
LSOA: Lower Super Output Area
NICE: National Institute for Health and Care Excellence
PAF: Postcode address file
PTSD: Post Traumatic Stress Disorder
UK: United Kingdom
WHO: World Health Organization
